# Liver proteome dataset of *Sparus aurata* exposed to low temperatures

**DOI:** 10.1016/j.dib.2019.104419

**Published:** 2019-08-19

**Authors:** S. Ghisaura, R. Melis, G. Biosa, D. Pagnozzi, H. Slavski, S. Uzzau, R. Anedda, M.F. Addis

**Affiliations:** a Porto Conte Ricerche, SP 55 Porto Conte/Capo Caccia, Km 8.400, Loc. Tramariglio, 07041 Alghero, Italy; b Aller Aqua, Allervej 130, DK-6070, Christiansfeld, Denmark; c Università degli Studi di Sassari, Dipartimento di Scienze Biomediche, Viale S. Pietro 43/B, 07100 Sassari, Italy; d Università degli Studi di Milano, Dipartimento di Medicina Veterinaria, Via Celoria 10, 20133 Milano, Italy

**Keywords:** Cold stress, Gilthead sea bream, Low temperature exposure, Liver proteins, Shotgun proteomics, Liver proteomic dataset

## Abstract

We report the proteomic dataset of livers from *Sparus aurata* exposed to low temperature during growth. Gilthead sea bream juveniles were reared in Recirculating Aquaculture Systems (RAS) and exposed to a temperature ramp made of two phases of four weeks each: a Cooling phase from 18 °C (t0) to 11 °C (t1) and a Cold Maintenance phase at 11 °C (t1-t2) in a 8 week feeding trial. At the end of the experiment, sea bream livers were collected and analyzed with a shotgun proteomics approach based on filter-aided sample preparation followed by tandem mass spectrometry, peptide identification carried out using Sequest-HT as search engine within the Proteome Discoverer informatic platform, and label-free differential analysis.

The mass spectrometry data have been deposited to the ProteomeXchange Consortium via the PRIDE partner repository with the dataset identifier PXD011059 (Vizcaíno et al., 2016; Deutsch et al., 2017; Perez-Riverol et al., 2016). The dataset described here is also related to the research article entitled “Liver proteomics of gilthead sea bream (*Sparus aurata*) exposed to cold stress” (Ghisaura et al., 2019).

**Table:** Specifications Table

Subject area	*Biology*
More specific subject area	*Proteomics*
Type of data	Tables
How data was acquired	Q-TOF hybrid mass spectrometer equipped with a nano lock Z spray source and coupled on-line with a NanoAcquity chromatography system (Waters)
Data format	Raw, processed
Experimental factors	Proteome analysis of gilthead sea bream livers during exposure to low temperatures: Cooling phase from 18 °C (t0) to 11 °C (t1); Cold maintenance Phase at 11 °C (t2)
Experimental features	1) Protein extraction (mechanical disruption in TUC-based buffer) 2) Filter-aided sample preparation (FASP) 3) LC-MS/MS analysis
Data source location	Tramariglio, Alghero (SS), Italy; Torregrande (OR), Italy
Data accessibility	Data is within this article and available via the ProteomeXchange Consortium, dataset identifier PXD011059
Related research article	Ghisaura S, Pagnozzi D, Melis R, Biosa G, Slawski H, Uzzau S, Anedda R, Addis MF. Liver proteomics of gilthead sea bream (*Sparus aurata*) exposed to cold stress. J Therm Biol 2019; 82:234–41. https://doi.org/10.1016/J.JTHERBIO.2019.04.005. *Author names:* S.Ghisaura; D.Pagnozzi; R.Melis; G.Biosa; H.Slawski; S.Uzzau; R.Anedda; M.F.Addis; *Title:* Liver proteomics of gilthead sea bream (*Sparus aurata*) exposed to cold stress; *Journal:* Journal of Thermal Biology

**Table:** 

**Value of the data** • Detailed proteomic dataset of gilthead sea bream livers in fish exposed to low temperature during growth; • Differential protein abundances between the two temperature phases: Cooling phase (t0-t1, from 18 °C to 11 °C) and Cold Maintenance phase (t1-t2, at 11 °C) are useful to understand the dynamics and metabolic shifts occurring in sea bream liver with decreasing water temperature; • Shotgun proteomics dataset improves previous data on fish hepatic metabolism during cold exposure; • The proteomic dataset might be advantageous to other research groups working on the development of feeds designed to compensate the thermal stress encountered by fish in offshore farming conditions.

## 1 Data

Sea bream livers exposed to low temperatures (Cooling phase and Cold maintenance phase) were characterized with a shotgun proteomic approach. A summary of protein identifications obtained in all samples is provided in Table 1. All protein identifications obtained with the Proteome Discoverer software in gilthead seabream livers exposed to three temperature phases (t0, t1, t2) are listed in Supplementary Table 1.

**Table 1:** Summary of protein identifications obtained in all samples.

	*t0*	*t1*	*t2*
#Proteins	649	620	654
#Proteins with PSM ≥2	510	458	487
#Peptides	1491	1328	1448
# PSM(s)^a^	5176	4274	4837
#Search inputs^b^	19391	19025	20644
#Total Spectra^c^	22036	21607	23302

a The number of peptide spectrum matches obtained for protein identification.

b The number of preprocessed spectra by the Spectrum Selector node in the workflow used in Proteome Discoverer software (precursor mass range: 350–5000 Da; Signal/Noise Threshold: 1.5).

c The total number of spectra obtained by LC-MS/MS run.

The differential analysis was carried out with a label-free approach by comparing all different groups according to temperature variations: Cooling phase (t0-t1), Cold Maintenance phase (t1-t2) and Overall changes (t0-t2). The differential proteins passing the significance thresholds (R_NSAF_ > 0.5 or < −0.5; P value < 0.05; FDR < 0.1) are summarized in detail in the related research article [4]. Detailed protein identification and abundance data for the three comparisons (t0 vs t1; t1 vs t2 and t0 vs t2) are provided in Supplementary Table 1.

## 2 Experimental design, materials, and methods

### 2.1 *Sparus aurata* liver samples

Gilthead sea bream specimens with an average weight of 82.0 ± 4.5 g were selected for the experimental feeding trial. A total of 60 juveniles were transferred in three 550 L tanks and an acclimation phase of two weeks from 20 °C to 18 °C was carried out. Water temperature was gradually lowered as described in Ghisaura et al., 2019 [4]. During the trial, fish were fed with an experimental feed formulation (Aller Aqua, Christiansfeld, Denmark) by hand, once a day. After fish anesthetization with 1,1,1-trichloro-2-methylpropan-2-ol (2% in marine water) and transfer in a mixture of marine water and ice, liver tissues were collected at each time point (t0, t1, t2). The complete procedure of tissue excision and storage is described by Melis et al., 2017 [5].

### 2.2 Protein extraction and digestion

Liver tissues were subjected to protein extraction and quantification according to Ghisaura et al., 2016 [6]. All protein extracts were then subjected to on-filter reduction, alkylation, and trypsin digestion according to the filter-aided sample preparation (FASP) protocol [7], with some modifications [8]. Peptide mixture concentration was estimated by using the BCA protein assay kit (Thermo Scientific - Rockford, IL).

### 2.3 LC-MS/MS analysis

A Q-TOF hybrid mass spectrometer with a nano lock Z spray source, coupled with a NanoAcquity chromatography system (Waters) on-line, was used for LC–MS/MS analyses as described in Pagnozzi et al., 2014 [9]. LC-MS/MS procedures are fully described in Ghisaura et al., 2019 [4].

### 2.4 Data analysis

Proteome Discoverer software (version 1.4.0.288; Thermo Scientific) was used to analyze the peak lists from the Q-TOF instrument, after conversion into an MGF file. The workflow was as described in Ghisaura et al., 2019 [4]. Gene ontology and protein annotations were retrieved from UniProtKB (http://www.uniprot.org). The uncharacterized sequences were identified by homology through blasting on NCBI as another non-redundant database (http://blast.ncbi.nlm.nih.gov/Blast.cgi).

Differential protein abundances of different functional categories (Cooling phase; Cold maintenance phase; and Overall changes) were estimated by the Normalized Spectral Abundance Factor (NSAF) according to Zybailov et al., 2006 [10]. The significance threshold RNSAF >0.5 or < −0.5 was applied for analysis. To evaluate the statistical significance of differential protein abundance between logarithmized (normally distributed) NSAF values, a student's t-test (two-sample comparison, p < 0.05) was applied. Logarithmized NSAF values were furthermore corrected by using a false discovery rate (FDR) as a multiple hypothesis testing, with FDR < 0.1 as a threshold limit. The dataset was then deposited in the ProteomeXchange Consortium via the PRIDE partner repository (identifier PXD011059) [1], [2], [3], [11].
